# Making salt-restricted diets work for older adults: a philippine nursing perspective

**DOI:** 10.1016/j.tjfa.2026.100228

**Published:** 2026-11-05

**Authors:** Erella Palo, Bella G. Fontanilla, Kenneth Jhon Babaran, MaryJane Gonzales

**Affiliations:** College of Nursing, Public Health and Midwifery, University of La Salette, Santiago City, Philippines

Dear Editor,

We read with great interest the article “This has no flavor”: balancing nutrition, taste, and salt restriction in geriatric care units by Marzetti et al. The article identifies a clinically important tension: sodium restriction may be appropriate for selected older adults, yet a meal that is perceived as bland may be consumed in insufficient quantity. We extend this discussion by situating the problem more explicitly in Philippine nursing practice, where nutritional inadequacy, culturally familiar eating patterns, and practical monitoring of meal intake are important considerations [1].

The Philippine context is particularly relevant because inadequate dietary intake is already documented at the population level. Analysis of the 2013 Philippines National Nutrition Survey included 7,853 adults aged 50 years and older and found high prevalences of inadequate intake of several nutrients; energy inadequacy was also substantial across the adult groups. The authors identified a diet dominated by refined rice and relatively low contributions from vegetables, fruits, and dairy foods. These findings do not establish that sodium restriction causes malnutrition, but they demonstrate that many Filipino adults enter later life with limited nutritional margins. In this setting, a hospital diet that further reduces food acceptance may be clinically consequential for an older person who is already eating poorly [2].

Philippine evidence is also directly relevant to palatability. Bautista and colleagues conducted a crossover study among older persons at Golden Acres Institution in Quezon City. Their 2013 study included 60 participants aged 60 years and older and found that flavor-enhancer interventions increased energy and most nutrient intakes; body weight and body mass index also increased significantly during the monosodium glutamate intervention. Importantly, however, this study should not be interpreted as evidence that sodium-containing flavor enhancers are appropriate for patients who require therapeutic sodium restriction. The participants were not a population receiving a prescribed low-sodium diet, and the intervention itself included iodized salt in one treatment condition. The more useful lesson for geriatric nursing is that palatability can influence intake and therefore deserves assessment alongside the prescription itself [3].

This distinction is especially important in the Philippines, where food is not simply a source of nutrients but also a familiar part of daily routines and family life. Nurses caring for older adults should therefore avoid equating “low sodium” with “bland.” Taste preference is not necessarily a direct indicator of sodium consumption, and dietary sodium restriction can alter taste responses to sodium chloride, underscoring the importance of assessing individual food acceptability [4,5] When clinically appropriate, meal planning can preserve sensory appeal through culturally familiar ingredients and preparation techniques that do not depend primarily on added salt—for example, garlic, onion, ginger, herbs, spices, and acidic ingredients such as calamansi or vinegar. Philippine National Nutrition Council guidance similarly recommends using herbs and spices as alternatives to added salt for older adults [6]. Adapt these approaches to the individual's swallowing ability, oral health, renal function, cardiovascular condition, medications, and prescribed sodium target.

The nursing role is therefore more specific than simply reinforcing a dietary order. At admission and during hospitalization, nurses can document appetite, percentage of meals consumed, changes in taste or smell, chewing or swallowing difficulty, oral discomfort, food preferences, and reasons for leaving meals unfinished. A persistent decline in intake should trigger timely interdisciplinary review with the physician, dietitian or nutrition professional, and, when indicated, a speech and swallowing specialist. For patients at nutritional risk, the question should not only be “Is the sodium restriction being followed?” but also “Is the patient eating enough of the food provided?” This is consistent with the person-centered approach advocated in geriatric nutrition guidance, which emphasizes individualized nutritional and hydration care [7].

The Philippine healthcare context also supports a pragmatic nursing approach. Nutrition assessment is often performed at the bedside, making nurses central to recognizing the mismatch between a prescribed diet and actual intake. Simple repeated observations—such as meal consumption, appetite, swallowing tolerance, and patient-reported acceptability—can provide clinically useful information for the care team. Where specialist nutritional services are available, nurses can communicate these observations early so that sodium targets and nutritional goals can be balanced rather than managed as separate priorities.

We also suggest that future Philippine research move beyond whether salt should be restricted and examine how older patients experience restrictions. Studies in hospitals and long-term-care settings could measure prescribed sodium targets, actual meal consumption, dietary energy and protein intake, weight change, nutritional-risk scores, food acceptability, and functional outcomes. Research should also distinguish patients for whom sodium restriction is strongly indicated from those for whom less restrictive dietary strategies may be clinically acceptable. This would provide evidence for nursing protocols that are both nutritionally safe and culturally responsive.

In conclusion, Marzetti et al.'s concern is particularly relevant to Philippine geriatric nursing. The issue is not whether sodium restriction is necessary when clinically indicated, but whether the prescribed diet is palatable enough for an older person to eat adequately. Philippine evidence on nutritional inadequacy and flavor enhancement suggests that palatability deserves a more explicit place in nutritional assessment. A culturally responsive, person-centered approach—supported by nurses and the wider interdisciplinary team—may help ensure that therapeutic dietary restrictions do not unintentionally undermine nutritional adequacy, function, and quality of life [[1], [2], [3]].

## Funding

The authors received no funding for this work.

## Conflict of interest

The authors declare no conflict of interest.

## Declaration of AI use

The authors used generative artificial intelligence only for language editing and manuscript formatting. The authors reviewed and take full responsibility for the final content.

## CRediT authorship contribution statement

**Erella Palo:** Validation, Conceptualization. **Bella G. Fontanilla:** Supervision. **Kenneth Jhon Babaran:** Supervision. **MaryJane Gonzales:** Conceptualization.
